# Integrating natural gradients and controlled assays to reveal bacterial responses to cadmium in *Theobroma cacao* L., soils

**DOI:** 10.1371/journal.pone.0345645

**Published:** 2026-03-24

**Authors:** Claudia Jaramillo-Mazo, Daniel Bravo, Diego Fernando Villanueva-Mejía, Javier Correa-Alvarez

**Affiliations:** 1 Research Group in Biosciences and Biotechnology (TechLife), School of Applied Sciences and Engineering, EAFIT University, Medellín, Colombia; 2 Research Group in Agrobiotechnology, Faculty of Exact and Natural Sciences, Universidad de Antioquia, Medellín, Colombia; 3 Laboratory of Soil Microbiology & Calorimetry. C.I. Tibaitatá. Corporación Colombiana de Investigación Agropecuaria AGROSAVIA, Mosquera, Colombia; Universidade de Coimbra, PORTUGAL

## Abstract

Cadmium (Cd), a toxic heavy metal found in agricultural landscapes worldwide, has been pointed out in cropped soils with *Theobroma cacao* L., as one of the main contaminants that translocate into plant tissues. Among the factors linked to cadmium translocation into plants, the role of soil bacterial communities in chemical transformation in soils has been poorly investigated. Overall, soil bacterial communities are shaped by diverse environmental and anthropogenic factors that influence crop yield and health. Cadmium alters soil microbial communities and increases the risk to human health through plant uptake. Although the impacts of cadmium on soil bacteria have been studied in other crops, there is limited information on cacao. Thus, this study aimed to assess the responses of soil bacterial communities in cacao farms to cadmium exposure, both natural and spiked. A total of 225 rhizosphere soil samples were collected from 16 plots across five cacao farms in two Colombian departments. The complementary approaches used were: (i) 16S rDNA amplicon sequencing to assess the composition of the bacterial community in soils with natural Cd concentrations, and (ii) isothermal microcalorimetry (IMC) to measure the temporal metabolic responses of bacteria to Cd in closed systems for 80 hours at 25 °C. The findings suggest that nearly 28% of the bacterial community responds to high cadmium concentrations in soils, both in natural and experimental conditions. Field-based observations revealed that Cd-responsive taxa detected under natural soil conditions included several unculturable bacterial groups, whereas laboratory experiments with Cd spiking predominantly selected for previously characterized cadmium-tolerant bacteria (CdtB). Significant variation in natural Cd-bacterial community composition and Cd-related metabolic activity was observed across the farms. Moreover, Cd-responsive bacterial taxa exhibited increased abundance during Cd spikes. As expected, contrasting patterns were revealed by the activity-response measured by IMC and taxonomic analyses of 16S rRNA gene sequences.

## Introduction

Cadmium (Cd) is a heavy metal commonly found in agricultural soils worldwide. It can negatively affect soil health and increase heterogeneity [1–4], thereby impacting the safety of crops for human consumption. Its primary impact is associated with Cd translocation into different cacao plant tissues [1,5–7]. This process is influenced by various factors, including the composition of the soil microbial community [8–12]. The presence of heavy metals such as Cd in soils creates unbalanced conditions for plants and microorganisms alike, altering nutrient availability and uptake as well as microbial niche dynamics [13–16]. Studies on the effects of Cd on soil bacterial communities in crops such as *Oryza sativa* L. [17], *Arabidopsis helleri* [18], and soybean [14], have demonstrated that varying Cd concentrations can impose selective pressure on microbial biomass [16,19–21] and alter bacterial community structure and diversity [22].

In *Theobroma cacao* L., a tropical crop of high economic and nutritional importance, some progress has been made in understanding soil bacterial communities, primarily in the context of agroforestry management [23] and organic cocoa production systems [24]. However, there are few reports on bacterial communities associated with cadmium-contaminated cacao soils [12,25–28], despite cacao’s importance for human consumption [29]. While there is evidence that microbial communities contribute to soil processes [30], and that bacterial community structure and diversity are modulated by nutrient interchange under intercropping conditions [14], little is known about how soil microorganisms in cacao crops respond to Cd contamination [28,31]. Despite the known role of microbial communities in Cd translocation to cacao plants [12,26], fewer studies have examined these responses under natural conditions.

Agricultural properties, such as soil pH [10], soil organic matter (SOM) [32], and landscape, including environmental and anthropogenic factors [6,33], have been associated with the composition of soil bacterial communities [34]. These associations may result in changes to crop yield and health. Indeed, to develop healthier crop management strategies, it is particularly important to assess how microbiomes assemble, maintain fertility, and respond to agronomic conditions. This is especially important given that microbial communities can vary considerably even at micrometer or millimeter scales within soil [30,35,36].

Although it is expected that different bacterial taxa will modulate microbial Cd-related activity [37,38], it remains unclear what proportion of soil bacterial communities from different geographical origins respond to Cd under natural and experimental conditions. Ultimately, the choice of bioremediation strategy will depend on the risk management assessment of the selected agroecosystem [39]. To understand Cd dynamics within the microbial community in cacao growing soils, techniques such as 16S rDNA amplicon sequencing [40] and isothermal microcalorimetry (IMC) are useful as they allow evaluation of both soil microbial composition and microbial metabolic activity through richness indexes and heat production rates [41,42], even with non-culturable microorganisms.

Henceforth, to assess both the microbiome diversity and metabolic activity of the microbial community, two approaches are highlighted here as particularly useful. One way is by the 16S rDNA amplicon sequencing to characterize community composition [12]. In another way, performing an isothermal microcalorimetry (IMC) assay using soil [43], where DNA is also extracted, to assess soil Cd-related bacteria metabolic activity. Specifically, our aims were: (i) to evaluate the composition of soil bacterial communities and their response to natural Cd concentrations in different geographical locations, and (ii) to measure the response of bacterial communities to Cd *in vitro,* both with and without Cd spiking. The former approach shows the composition of the entire bacterial community according to natural Cd conditions in cacao-growing soil at a specific point in time in the calorimetric assay, as determined by soil samples. In contrast, the latter approach provided an overview of bacterial Cd-related activity of the same soil samples using thermal monitoring through time, enabling comparison between the original community composition, its response to Cd spiking, and the final bacterial community composition when the experiment ended. The overall bacterial community response to increased Cd content is, therefore, described. The soil Cd content in the sampled farms primarily reflects natural geological variation from Cd-rich parent materials commonly found in Colombian volcanic and sedimentary soils. However, anthropogenic inputs, including phosphate-based fertilizers and contaminated irrigation water, may contribute to elevated Cd levels in certain agricultural zones [44]. The farms selected for this study represent a gradient of predominantly natural Cd concentrations, enabling assessment of bacterial community responses across environmentally relevant exposure levels.

This study hypothesizes that soil bacterial communities would respond variably to farm-specific conditions, and that contrasting patterns would be revealed by taxonomic (16S rRNA) and activity-response profiles in soils with and without cadmium (Cd) spiking. Furthermore, we expected Cd exposure to promote shifts in community composition over time, favouring Cd-tolerant taxa. To test this, we compared 16S rRNA amplicon sequencing with measurements of natural Cd concentrations in 225 rhizosphere soil samples collected from sixteen plots across five cacao farms in two Colombian departments.

## Methods

### Soil sampling in cacao plants and DNA extraction of bacterial microbiome from soils

The soil samples collected for this study were taken from cacao farms in the departments of Antioquia and Santander in Colombia during 2020 and 2022. The samples from Antioquia were taken from two different geographical locations: Urabá and Magdalena subregions. The samples from Santander were taken in the municipality of San Vicente de Chucurí. Rhizosphere soil samples were collected from 225 individual cacao plants distributed across 16 plots within five farms. To each plot, 14−20 plants were sampled depending on plot size. To each plant, it was considered for sampling the rhizosphere, as the area adjacent to the roots. Thus, the rhizosphere soil samples were collected at 0−20 cm depth, targeting the zone of highest root density. For each plant, three subsamples were collected from equidistant points around the trunk and thoroughly mixed in the field into a composite homogenous sample per plant for an average Cd concentration measurement and bacterial community composition analysis. Physicochemical soil parameters were measured for each sampling plot in every plantation, mixing all the soil samples per plot into a composite sample (n = 16). Details of the farmland locations and selection can be found in a recent study [12]. The samples were transported and stored at 4 °C until processing. The soil samples were air-dried and sieved through a 60-mesh sieve (Sigma Aldrich, Darmstadt, Germany) to remove rocks, invertebrates, or leaves, in preparation for the bacterial DNA extraction process. According to the manufacturer’s instructions, DNA was extracted from 250 mg of soil per sample using the DNeasy Powersoil Pro Kit (QIAGEN, Germantown, MD, USA). DNA integrity and concentration were assessed by gel electrophoresis at a 1.2% agarose concentration and measured using both the Nanodrop™ spectrophotometer (Thermo Fisher Scientific, USA) and a Qubit® 3.0 Fluorometer (ThermoScientific, USA). Total DNA was then amplified using a polymerase chain reaction (PCR) for rDNA 16S with standard primers 515F/806R [45]. Libraries were created using paired short reads of 300 bp from the rDNA 16S V3-V4 region, followed by sequencing on an Illumina MiSeq platform [46] at the University of Massachusetts Dartmouth facility (USA). The 16S rDNA gene sequences obtained in this study were stored in the National Center for Biotechnology Information Sequence Read Archive (Accession ID: PRJNA1015591). The study was carried out following local and national guidelines for fieldwork; hence, field sampling was performed with the legal permits according to the National Environmental Licensing Authority, ANLA (Medellín, Colombia) (N°2021047621-1-000; 2021047841-1-000 and 2021047842-1-000). The sampling collection was designed to minimize environmental disturbance. The ethics committee of EAFIT University approved this study.

### Reads processing, and ASV table assignation

To characterize the bacterial community composition of all the samples and sites mentioned, the paired-end reads were processed further for error correction and to generate amplicon sequence variants (ASVs) using the DADA2 module in the Qiime2 package pipeline [47,48]. Each ASV was then annotated taxonomically using the q2-feature-classifier module, which was trained using the SILVA reference database (v. 132) [49]. Singletons and non-target reads were removed to avoid potential contaminants. The data were organized with phyloseq (Bioconductor v.3.15), with species-level sequence identification performed at 100% identity. The ASV table was proofread to ensure an equal number of sequences per sample, enabling comparisons between samples.

### Statistical analysis of soil bacterial communities and definition of Cd-response

Analyses of the soil microbiome were carried out using RStudio v4.1.0 (https://www.r-project.org/) and the following main packages: *qiime2R* [48], *phyloseq* [50], *vegan* [51], *Ancombc* [52], *ggplot*2 [53], *BiocManager* [54], *Microbiome* [55], and *VennDiagram* [56]. It was assumed that the bacteria responding to different natural Cd concentrations in all the selected farms were Cd-responsive bacteria, based on their composition. Meanwhile, the Cd-responsive bacteria, based on their activity and composition, were differentially abundant under experimental conditions compared to the control (without spiking). Cd-responsive bacterial community analysis was performed on all samples collected from five farms with different Cd soil concentrations, using ANCOMBC to compare the bacterial community composition under low and high Cd amounts in soils. Similarly, the analysis of Cd-responsive activity and composition of bacterial community was carried out on soil samples with and without Cd spiking under experimental conditions. Alpha-diversity indices (species richness, *Chao1*, and *Shannon* diversity) were calculated for each sample and clustered by agronomic units (farms). Differences in alpha diversity among farms were assessed using the Kruskal–Wallis test followed by Dunn’s post hoc comparisons with Benjamini–Hochberg correction. Groups sharing the same letter are not significantly different (*p > 0.05*). We used nonmetric multidimensional scaling [51] with Bray-Curtis distances to analyze spatial patterns in community composition, and *vegdist* was used to identify the factors explaining the variation in these patterns [12]. Additionally, we performed a PERMANOVA [57] with 999 permutations using the *Adonis* function in the *vegan* package. The dissimilarity of taxonomic composition was compared with the soil Cd content for all sampled soils. Community dispersion of ASVs was studied based on Bray-Curtis distances using a canonical analysis of principal coordinates (CAP) to infer hypotheses relating to communities’ response to environmental conditions or taxonomic patterns in accordance with soil Cd concentration. ANCOM-BC analysis was performed on the bacterial community analysis of Cd-spiking samples to detect ASVs with differential abundances in the control and treatment samples. These were then compared to identify the taxonomic groups present only in the Cd-spiking samples (amended with 1 mg L ⁻ ¹). Furthermore, heatmaps were created using the *plot_heatmap* function in the *phyloseq* package [50]. These heatmaps were based on differential abundance and included taxa detected only in Cd-spiked samples from each farm to demonstrate the gradient generated between subsample moments during the experiment (starting = S, peak activity detected = P, and ending = E). Venn diagrams were used to identify ASVs that were shared or unique to the three Cd categories evaluated, using the *venn* function in the *eulerr* package with a prevalence/detection threshold of 0.001/100.

### Isothermal Microcalorimetry (IMC) assay and Cd-responsive bacteria identification

**Fig 1:**
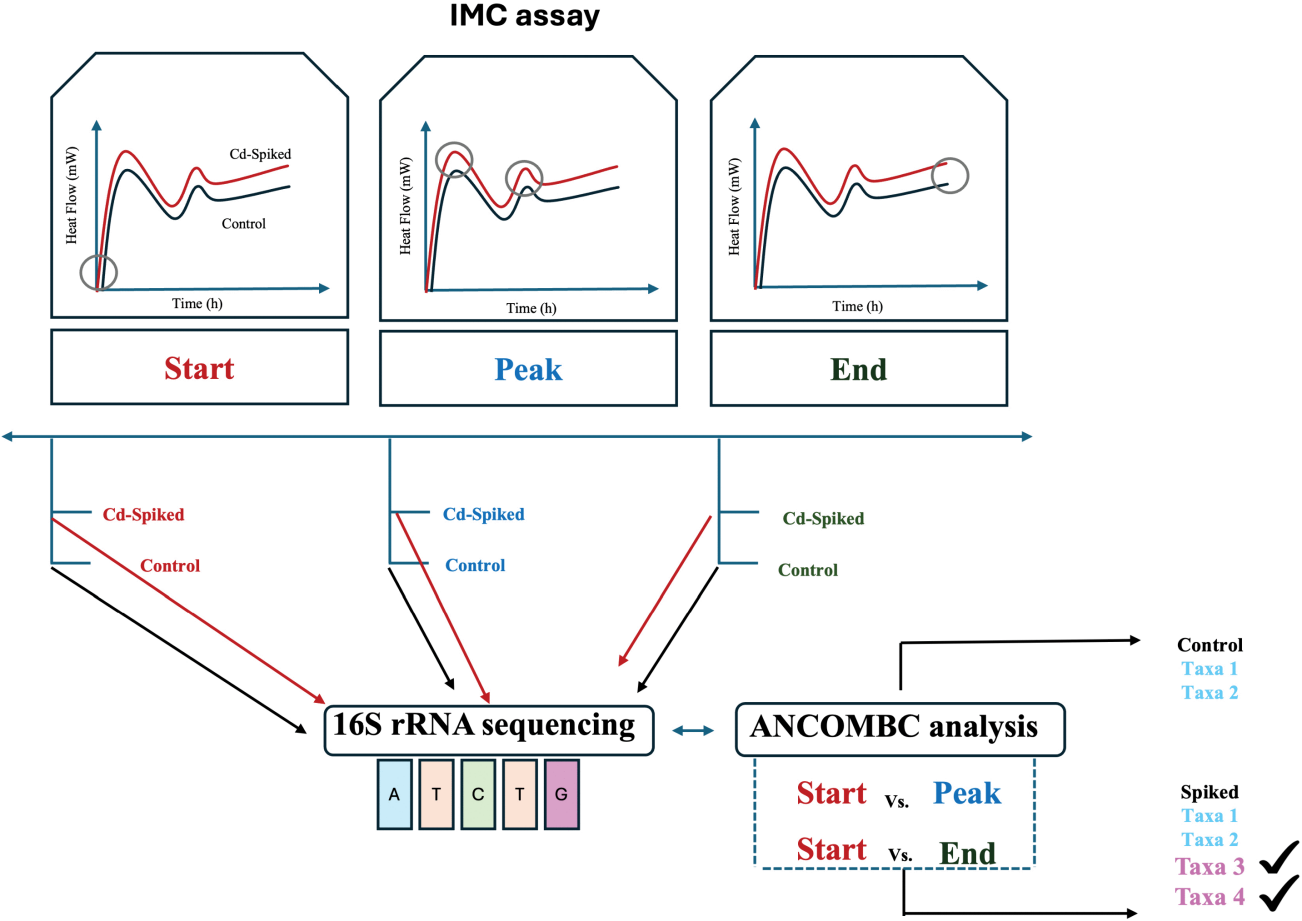
A flowchart describing the isothermal microcalorimetry assay carried out to identify Cd-responsive bacteria. This approach integrates the combination of IMC assays and 16S rRNA sequencing to identify Cd-responsive taxa. For each sample, the heat flow within a spiked sample and a control was measured. The difference in heat flow between the spiked sample and the control (Δ heat flow) represents the activity related to Cd amendment. During the experiment, the 16S rRNA was analysed at the start, during the peaks, and at the end of the assay, in both control and spiked samples. Using ANCOMBC, it was identified the taxa that were enriched during the peaks and at the end, compared to the control. Cd-responsive bacteria were defined as taxa enriched in Cd-spiked samples that were not enriched in their corresponding control samples.

Isothermal microcalorimetry assay (Fig 1) was performed for 60 soil samples from the five farmlands (6 samples per farm) described above. We aimed to continuously measure the change in heat production (i.e., activity) using IMC on each sample for 80 hours. The novelty of our approach lies in the assessment of each soil sample with and without Cd spiking (30 spiked samples and 30 control samples). Thus, each control sample was used as an activity baseline of the soil bacterial community, which was compared to the corresponding Cd-spiked sample. We assumed that when the difference in the heat production between each spiked sample and its respective control (i.e., delta heat flow) was over zero, the activity in the sample was due to Cd-responsive bacteria. In addition, we assessed bacterial community composition in each sample at the start (T0), at the peak (in case it occurred), and at the end of the 80 hours of the IMC assay (129 samples in total). For each control sample, we identified those taxa that were enriched during the experiment using the ANCOMBC approach. We anticipated that the increase in abundance of these control-enriched taxa during the IMC experiment could not be attributed to Cd (non-response bacteria). Likewise, we repeated the same ANCOMBC analysis in the Cd-spiked samples, where we expected to find both non-response and Cd-responsive bacteria. Finally, we identified Cd-responsive bacteria as taxa that were enriched in Cd-spiked samples but were not in the respective control samples.

The calorimetric analyses were performed using an 8-channel TAM Air instrument (TA Instruments/Waters, Delaware, USA). The soil samples were sieved to remove particles under the same conditions as those used for molecular testing. The only difference was that the samples were not dried, avoiding any loss of metabolic activity of bacteria. One gram of soil was diluted in 20 mL glass ampoules containing 3 mL of the selective Mergeay culture medium [58]. This medium was used to assess cadmium-tolerant bacteria as previously reported [28]. The choice of Mergeay media is of major importance to define Cd-tolerance, analyzing metallophilic, metal-resistant, and metal-tolerant microorganisms [59]. The Mergeay media [58] was designed to mimic polluted environments such as soil that may contain up to 10% Cd and still provide viable counts of heterotrophic Cd-tolerant bacteria. The IMC assay used paired comparisons within each soil sample: aliquots from the same sample were either left unamended as a control or spiked with 1 mg L ⁻ ¹ CdCl₂ (Sigma Aldrich, IL, United States) in the selective media. This paired design controls for baseline differences in microbial community composition and organic matter content between samples. Another control consisted of a soil sample, coming from a farm with the lowest soil Cd content (0.01 mg kg^-1^ on average). The calorimeter baseline was performed with ampoules containing sterile Mergeay medium with the same Cd concentrations as the inoculated ampoules. Therefore, the microbial metabolic activity was recorded by the automatic subtraction that the calorimeters do from the chemical reactions of the ampoules used as blank with the measured ampoules with the treatments, using the two-step thermal equilibration procedure [43,60]. The thermal monitoring conditions included constant agitation at 250 rpm using the Admix Ampoule accessories (TA Instruments, Delaware, United States; see https://www.tainstruments.com/wp-content/uploads/TAM-AIR-brochure.pdf) coupled to the Tam Air equipment. The Cd spiking was carried out to favor the proliferation of CdtB populations. Thus, the soil samples within the liquid media were stirred throughout the calorimetric experiment. The experiment was conducted at a temperature of 25 °C for 80 h to account for the heat flow signal from fast- and slow-growing bacterial communities, including CdtB populations. A subsample of each ampoule was taken at three monitoring points: at the start (S), after 37 h or when the peak of activity occurred (P), and at the end of the assay (E). Once extracted, the subsamples were stored at −20 °C for later DNA extraction.

### Analysis of IMC

The data obtained from the IMC experiment were used to convert the recorded heat flow rate, measured in milliwatts (mW) into heat, in Joules (J). The mathematical Gompertz equation [43, 60] was then used to fit the raw data to calculate biological processes. To this, an R script was used to determine the thermodynamic parameters such as (i) the maximum heat Q_max_, (ii) the time to achieve the maximum peak of activity TTP, and (iii) the Cd immobilization ratio Cd_imm_. Once determined, the data were used to calculate the kinetic parameters: (i). the adaptation phase λ, (ii) the growth rate µ, and (iii) the maximal growth rate µ_max_ [43,61]. This analysis considers the presence of these parameters of the microbiome in soil samples, where multiple populations and microbial communities produce a higher heat signal than isolated populations. It is worth mentioning that Cd spiking served to maximize the metabolic response of CdtB, particularly by stressing bacterial responses to higher concentrations of Cd than to natural exposure to the pollutant in cacao soils.

## Results

### The composition of the soil bacteria community in the field presented a large variation in alpha and beta diversity

Illumina sequencing allowed us to generate approximately 16 million paired-end reads of around 260 base pairs (bp) each. This was achieved by truncating the forward and reverse read lengths to 230 and 200 bp, respectively, to exclude low-quality reads from the analysis. Using DADA2 [47] within *Qiime2* [48], we obtained between 2788 and 25083 non-chimeric denoised sequences per sample and 49019 Amplicon Sequences Variant (ASVs) in total with a mean length of 292 bp.

**Fig 2:**
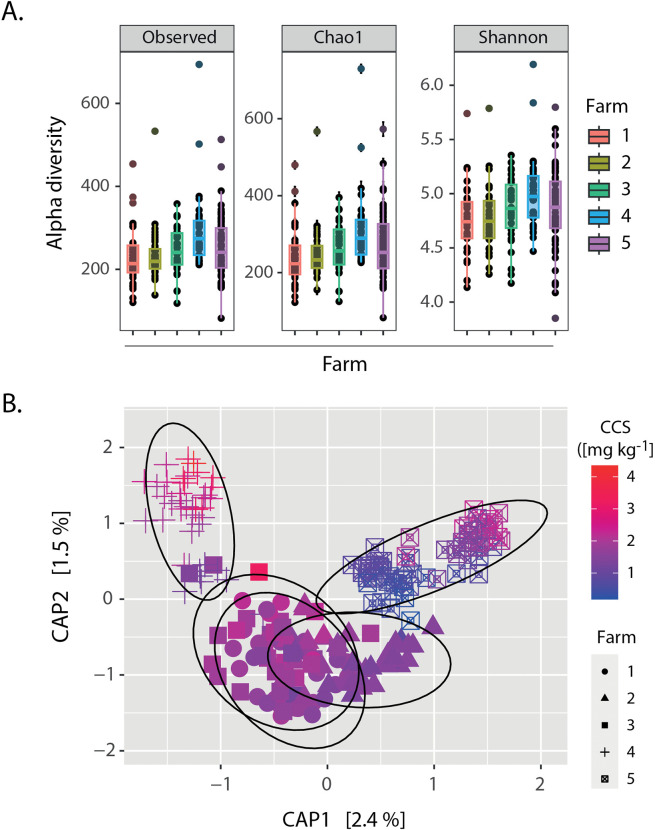
(A). Alpha diversity (Richness, Chao1, Shannon) for each farm. Colors and shapes represent a different farm. Different letters indicate significant differences based on Dunn’s post hoc test with Benjamini–Hochberg correction (*p < 0.05*). Farms sharing the same letter do not differ significantly. Observed richness Farm1: “a”, Farm2: “a”, Farm3: “a”, Farm4: “b”, Farm5: “a”. Shannon diversity Farm1: “a”, Farm2: “a”, Farm3: “ab”, Farm4: “b”, Farm5: “ab”. Chao1 diversity Farm1: “a”, Farm2: “a”, Farm3: “a”, Farm4: “b”, Farm5: “a”. **(B).** Canonical analysis of principal coordinates (CAP) *p < 0.001*, correlating soil bacterial community composition with Cd soil concentration and farms as an environmental factor. Shapes represent different farms, and the color gradient represents the natural Cadmium concentrations.

The alpha diversity **(**Fig 2A**)** of bacterial and archaeal communities in all soil samples (Species richness and Chao1-Shannon diversity) revealed that observed richness and Chao1 are significantly different in bacterial richness among cacao farms, with farm 4 differing from all other farms, while farms 1, 2, 3, and 5 did not differ from each other. In contrast, Shannon diversity showed fewer significant differences, indicating that changes in alpha diversity were primarily driven by richness rather than evenness.

The beta diversity of bacteria based on soil Cd and the farms was assessed using a canonical analysis of principal coordinates (CAP) **(**Fig 2B**)**. In axis 1 of the ordination, a discernible gradient emerged, indicating a separation between samples from different farms, whereas axis 2 shows a separation between samples with different Cd levels, especially in farms 4 and 5. Finally, the CAP model also revealed that both Cd soil concentration and farms significantly influence soil bacterial community composition (*p <* 0.001, PERMANOVA_Cd/FARM_ *p* < 0.05, PERMANOVA_Cd/PLOT_ *p* < 0.001). These findings highlight that the composition of the bacterial community responds not only to the natural amount of Cd in the soil, but also to the considerable variation in sampling location across different sites, which could be related to the variation in soils’ physicochemical conditions between sampling points within each farm location **(**S3 Table**)** [12].

### Soil core bacteria across different soil Cd categories

The data revealed differences in ASVs, both unique and shared, between soil Cd categories. The categories were: low (0−1 mg kg^-1^), medium (1−2 mg kg^-1^), and high (2–4.3 mg kg^-1^) Cd in soil content. Regardless of the distance between sampled farms, some soil core bacteria were detected in the soils of all five sites (S1A Fig); likewise, some soil core bacteria were detected in the soils of all Cd categories (S1B Fig). In the last, a total of 386 ASVs were shared between soil Cd categories, corresponding to those taxa that persist independently of any soil Cd concentration. Concerning ASVs found in farms, 97 taxa cores were detected, indicating that these ASVs are present in all sampled farms, even when located in different departments. Because the highest ASV proportions detected were unique within the categories or farms, it was necessary to assess the real taxonomic groups with differential abundance, as previously reported [52]. ANCOM-BC was employed as a statistically robust method for differential abundance analysis that accounts for compositionality bias and sampling fraction variability. Taxa exhibiting structural zeros (i.e., those completely absent in one treatment group) were identified and excluded from the analysis to ensure valid statistical comparisons. The category of low soil Cd (0−1 mg kg^-1^) was used as a reference group in the analysis to compare with those samples in high soil Cd (2–4.3 mg kg^-1^). The results showed that thirteen taxa had differential abundance in the high soil Cd category at the phylum level. Six phyla had differential abundance with a positive Log fold change, while seven had differential abundance with a negative log fold change (S2 Fig).

This study provides an identification, in natural soil Cd concentrations, of 46 phyla, of which 28% correspond to taxa that respond to the category of high soil Cd and 71% that did not respond to this condition (S1 Table). Of those that responded with differential abundance, 13% increased their abundance, while 15% decreased them. Almost all taxa with positive differential abundances are represented in the ten most common phyla observed in the taxonomy bar per farm (S3 Fig). Meanwhile, as shown in (S5 Fig), the taxa that changed between the start and end of the isothermal microcalorimetry experiment corresponded to 27%, while 73% remained stable. Among the responsive taxa, there was a 16% increase in terms of differential abundance, whereas 11% decreased.

### Comparison of response to Cd in natural versus experimental conditions

**Fig 3:**
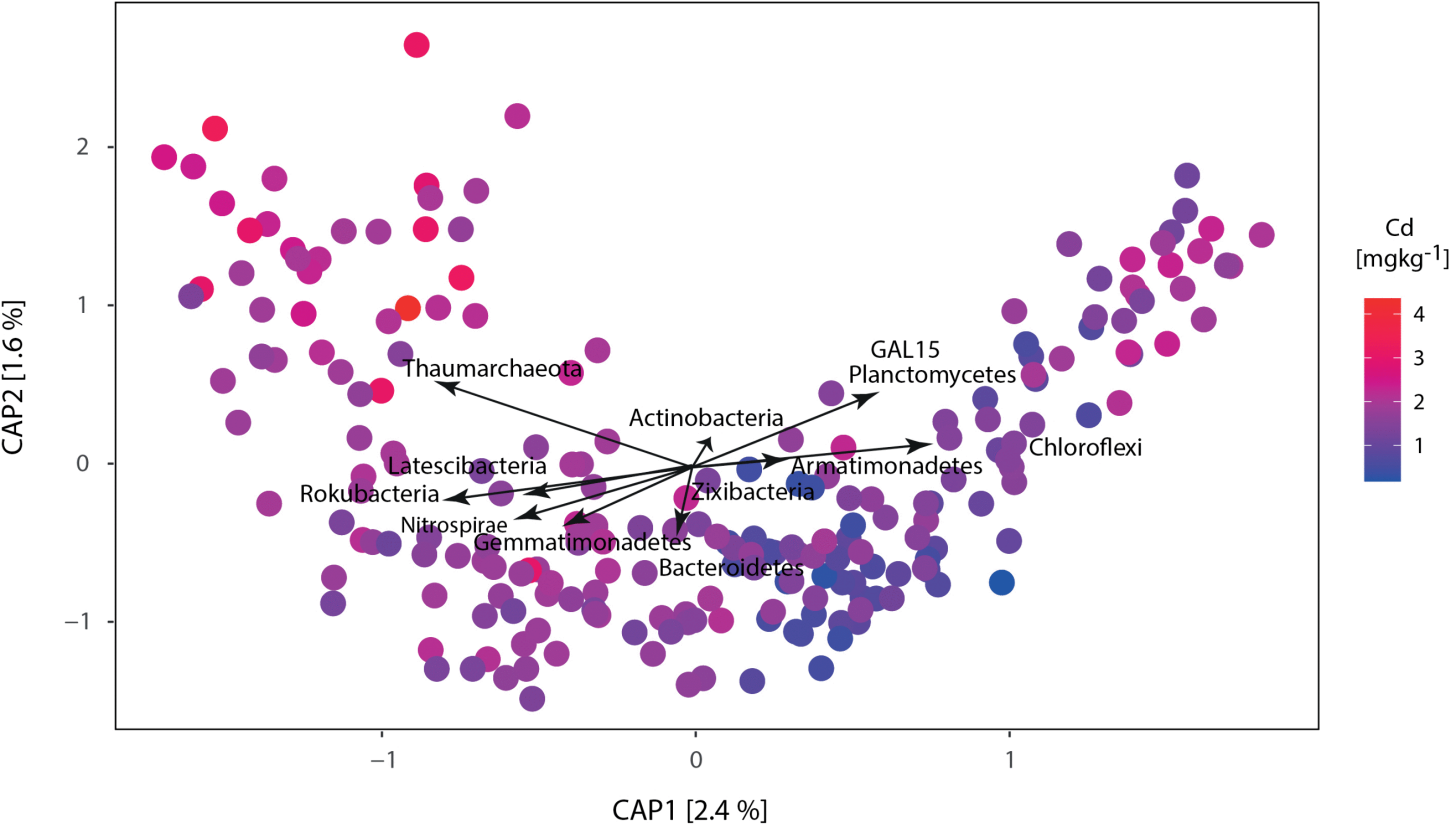
Canonical analysis of principal coordinates (CAP). The CAP shows the relationship between Cd_soil_ and the phyla that respond to this condition. The color gradient represents the natural Cadmium concentrations, and the arrows show the phyla associated.

The phyla in the collected samples that responded positively to the highest natural Cd concentration were *Thaumarchaeota, Gal15, Armatimonadetes, Actinobacteria, Chloroflexi*, and *Planctomycetes.* At the same time, those that negatively responded to Cd presence were *Bacteroidetes, Gemmatimonadetes, Latescibacteria, Nitrospirae, Rokubacteria*, and *Zixibacteria* (S1 Table). The analysis of principal coordinates ordination (Fig 3) shows the relationship between soil Cd and the taxa that respond to this condition, like the ANCOM-BC analysis, where the same phyla were detected as differentially abundant. The genera that best represent the phyla *Thaumarchaeota* and *Gal15* are uncultured bacteria, which underlines the challenge of assessing the role of Archaea and bacteria with the potential to react to Cd presence in cacao soil. In the experiment based on microbial response to Cd concentration, the main phyla that responded positively to the presence of Cd (compared to the control) were *Firmicutes*, *Bacteroidetes*, *Patescibacteria*, *Proteobacteria*, *Cyanobacteria,* and *Actinobacteria.* While those that negatively responded to Cd-spiking were *Chloroflexi, Acidobacteria, Planctomycetes,* and *Nitrospirae.*

### Soil sources modulated the Cd-response of bacterial activity

**Fig 4:**
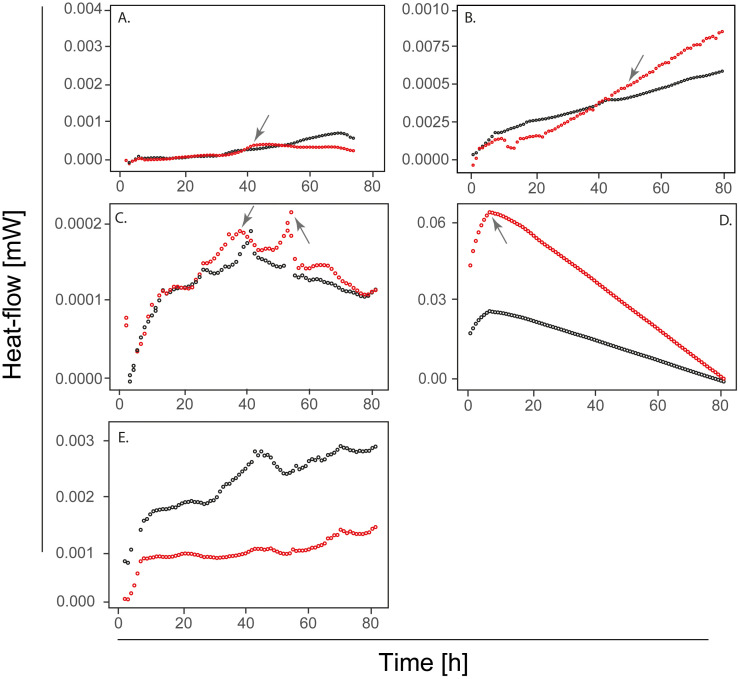
Thermograms of IMC showing the heat-flow (mW) of Cd metabolic activity in soil samples. Each thermogram represents one farm sample. **(A) (B)** (C) the heat-flow of soil samples from Farms 1, 2, and 3 in Urabá, Antioquia. **(D)** The heat-flow of the soil sample from Farm 4 in Santander. **(E)** The heat-flow of the soil sample from Farm 5 in Magdalena, Antioquia. Black lines correspond to samples with natural Cd content, while red lines show the metabolic activity of the corresponding sample amended with 1 mg L^−1^ of Cd.

**Fig 5:**
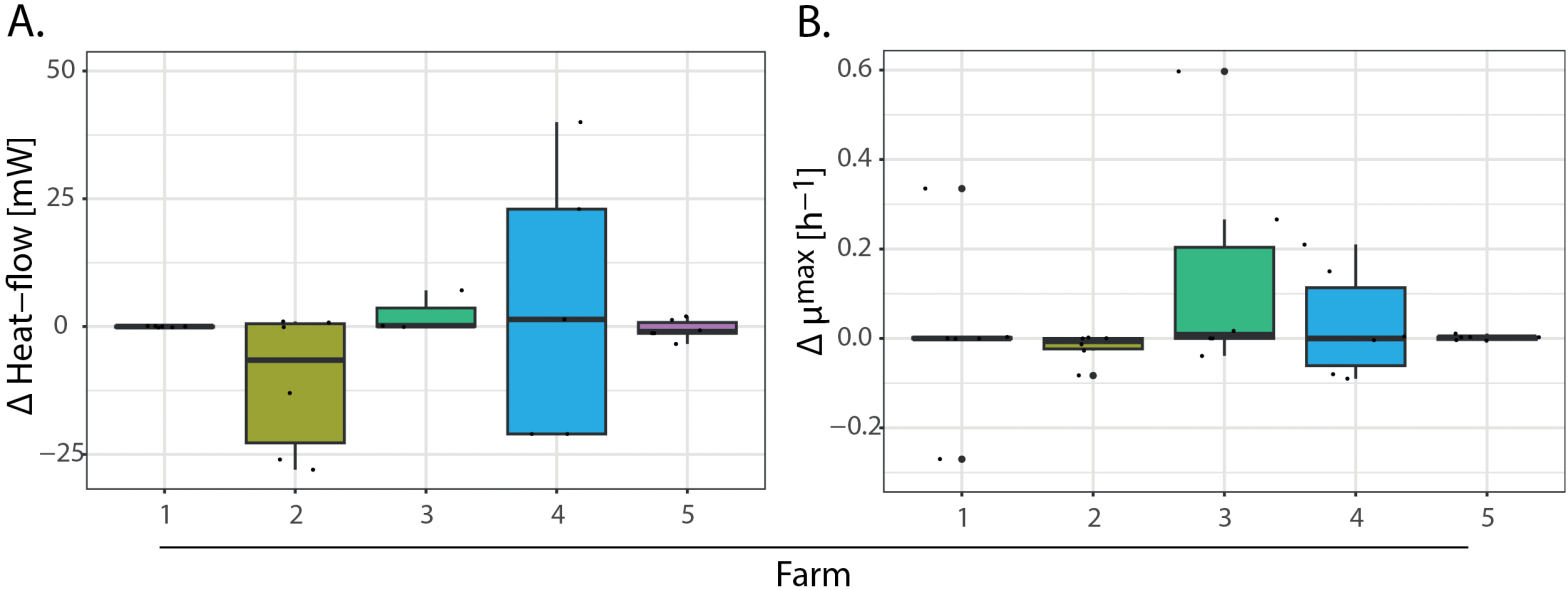
(A). The delta (Δ) of the heat flow of samples with 1 mg L^-1^ of CdCl_2_ to samples without.

The inspection of the patterns in the thermograms (Fig 4) and the thermodynamic parameters measured by IMC for all the samples (S2 Table) suggested that samples coming from different sources generate contrasting Cd-response activity patterns. The most representative IMC thermograms showed clear peaks of heat flow (i.e., metabolic activity) related to Cd metabolism (Fig 4C and 4D), like most of the samples in farms 3, 4, and 5. However, it is noted that the heat flow rates did not always increase continuously in samples spiked with 1 mg L^-1^ of Cd (Fig 4A, 4B) over their respective control sample, which was observed in 15 out of 30 total samples assessed. Likewise, in all farms, it was possible to observe some samples with more activity in their respective control (Fig 4E). It was assumed that samples with higher accumulated heat flow in spiked vs control samples had a metabolic activity related to Cd addition. Most of the samples from Farm 3 (Antioquia) and Farm 4 (Santander) showed that heat flow (mW) increased when spiked with 1 mg L^-1^ Cd, despite being farms in distant locations. Samples from Santander (**3**D as a representative one) showed the highest heat flow in the spiked soil sample. The maximum heat released (Q_max_) was variable between samples and farms (S2 Table), as was the delta (Δ) of the heat flow and the Δ of the growth rate (Fig 5). Samples from farms 3 and 5 presented positive Δ heat flow, highlighting that all these samples had consistently increased metabolic activity in spiked treatments compared to their counterparts without amendment. The Δ maximum growth rate (Fig 5B) coincides with the previously described findings in the Δ heat flow, where samples from farms 2 and 4 were the most variable. In both Δ’s calculated, samples from farm 1 were not significantly different between the treatments, spiked or not, showing isolated outliers.

amendment. Each color corresponds to the samples clustered by the farm. (B). The (Δ) maximum growth rate (μmax) of samples with 1 mg L^-1^ of CdCl_2_ compared to samples without amendment. Each color corresponds to the samples clustered by the farm.

## Discussion

### Large variation in cacao bacterial beta diversity is driven by sample origin and soil Cd concentration

The high dispersion of soil bacterial communities across farms and Cd natural concentrations was consistent across natural conditions and experimental approach (Fig 2B). This confirms that collection sites were highly heterogeneous in terms of soil Cd concentration, soil physicochemical parameters (S3 Table), and bacterial community composition, explaining the observed variation. As we showed in a previous work [12], the soil Cd content in these cacao farmlands was associated with physicochemical parameters such as K, Ca, CIC, soil Cd available, electrical conductivity, and carbon content. Despite this, it was shown that soil bacterial communities observed across farms were among the most important factors associated with Cd translocation from cacao soil to seeds.

It is well recognized that soil bacterial community composition is shaped by the interaction of multiple environmental drivers beyond Cd alone [30,62]. Nevertheless, multivariate analyses controlling for the farm effects (PERMANOVA_Cd/Farm_ *p* < 0.05), revealed a significant association between bacterial community structure and Cd concentration. Similarly, CAP analyses (*p < 0.001*) demonstrated that both Cd levels and farm location were significantly associated with variations in bacterial community composition. These results suggest that Cd exerts a detectable selective pressure on soil bacterial communities, even within highly complex and heterogeneous environmental settings.

As shown in Fig 2B, the different geographical locations of the farmland and the natural Cd gradient observed are relevant when assessing the response of the soil bacterial community to the presence of this heavy metal [12]. Likewise, the soil and plant variety characteristics are critical to understand Cd variations [63] across cacao farms [6,11]. This may play an important role in explaining the huge variability of bacterial diversity [35,64–66]. Previous studies have examined bacterial communities in cacao cropping systems, highlighting the influence of multiple interacting factors on their structure and function [26,65]. Research has shown that soil bacterial communities are linked to Cd translocation from soil to cacao seeds [12], while agroforestry systems [67,68], associated woody species [69], crop location [12,66], and agricultural management practices, including the use of Cd-contaminated fertilizers [44], further contribute to the heterogeneity of bacterial populations. Additionally, geographic location integrates environmental drivers such as altitude, climate, soil type, and mineral composition, which may act independently or synergistically to shape bacterial community composition [64,70].

Studies in heavy metal-contaminated agricultural soils have demonstrated that among location-dependent factors, soil pH consistently emerges as a primary driver of bacterial community structure [71], often acting through two mechanisms: direct physiological effects on bacterial cells and indirect effects by altering heavy metal bioavailability and speciation [70]. The sampled farms in this study spanned different departments in Colombia (Antioquia and Santander) with contrasting edaphoclimatic conditions, which likely contributed to the observed location-dependent patterns in bacterial community composition as observed in previous studies [28,72]. Identifying which of these location-dependent factors most strongly shape bacterial diversity, abundance, and functional activity in cacao soils represents an important avenue for future research.

### Non-culturable bacteria as a Cd-responsive taxa in cacao soils

Amplicon sequence variants (ASVs) aggregated by farm and soil Cd concentration, identified as the main drivers of bacterial community variability (S1 Fig), revealed consistent taxonomic patterns at the phylum level. (S3 Fig). Across farms, the most representative phyla included *Proteobacteria, Acidobacteria, Chloroflexi, Actinobacteria, Planctomycetes, Verrucomicrobia, Rokubacteria, Thaumarchaeota, Firmicutes*, and *Gemmatimonadetes*, with relative abundances varying among farms. These differences likely reflect the influence of soil type [73], physicochemical properties [35], plant species, and cacao varieties [6], as well as the natural soil Cd gradient. Differential abundance analysis (ANCOM-BC) showed that only a subset of taxa (*Actinobacteria, GAL15, Armatimonadetes, Thaumarchaeota, Chloroflexi*, and *Planctomycetes*) was significantly enriched at higher soil Cd concentrations. (S2 Fig). Although the presence of these taxa does not allow us to directly encompass bacterial activity in soil Cd mobilization and transformation, the same taxonomic classification of bacteria, even in distant farmlands, highlights their possible roles in soils contaminated with Cd. Notably, several of these phyla include predominantly uncultured lineages, underscoring the need for a more comprehensive understanding of their functional contributions, while others, such as *Actinobacteria, Chloroflexi*, and *Planctomycetes*, include reported Cd-tolerant representatives isolated from cacao systems [27,74].

### Thermodynamics vary between bacterial communities of different origins

The sensitivity of the IMC technique means that as few as 10⁴ CFU mL^-1^ metabolically active cells can be detected [41]. This technique has been used to monitor and quantify microbiological activity in a variety of applications, including soil microbial activity and contamination monitoring [75]. The thermodynamic parameters obtained by IMC varied between farms (S4 Fig). Heat flow variation was most prominent in three of the five farms: samples from farm 3 showed the highest heat flow, while samples from farm 1 showed the lowest. However, farm 1 samples exhibited the highest Q_max_ values. According to [12], farm 1 has minimal variation in soil Cd concentration but large variation in cacao beans’ Cd content. Notably, six samples (S02, S06, S09, S10, S20, and S22) exhibited the highest maximal growth rates (µ_max_) and maximum heat production (Q_max_) (S2 Table). Those samples came from farms 1 (Antioquia), 2 (Antioquia), and 4 (Santander).

The observed differences in responsive activity suggest that factors such as the initial soil bacterial composition could influence heat flow responses, with a major peak (the first 48 h) and a weaker one at the end (around 70 h) of the assay. Additionally, the natural soil Cd concentration per sample may also impact activity, as samples with metabolic activity during the experiment had Cd concentrations ranging from 1.5 to 3.13 mg kg^-1^.

Likewise, the heat flow during the experiments was relatively low in four of five farms compared to previous studies [43,72], which assessed soil samples for bacterial detection and cultivation under high activity conditions. This difference could be attributed to the shorter experimental duration in this study compared to the 288 h used by those authors. Despite the generally low heat flow, the Q_max_ found here confirmed the preexisting bacterial metabolic activity in the soil’s natural Cd contents. The slow response suggests that Cd’s impact on the bacterial community is less strong than that of other soil conditions (e.g., proximity to the rhizosphere) and that its effect is triggered at high Cd doses [16]. Said that, in soil samples spiked with soluble Cd exhibited the presence of peak delayed activity during the experiment (i.e., see the arrows in Fig 4) and a longer adaptation phase than controls, aligning with previous reports [43]. This could be related to the time required for Cd-responsive bacteria to activate their tolerance-metabolic activity. The negative Δ heat flow and Δ maximum growth rate (Fig 5A-5B) relative to the baseline may be caused by one of the following: i. metabolic activity linked to Cd processing was not stimulated, ii. The microbial communities present in the composite sample may have been metabolically dormant, or their activity was limited by available substrates, iii. certain chemical reactions in soil can be endothermic, absorbing heat and producing side effects and anabolic reactions [76]; or iv. it could be a negative activity response of the soil bacterial community to Cd spiking.

### Bacteria responded to Cd spiked in experimental conditions

**Fig 6:**
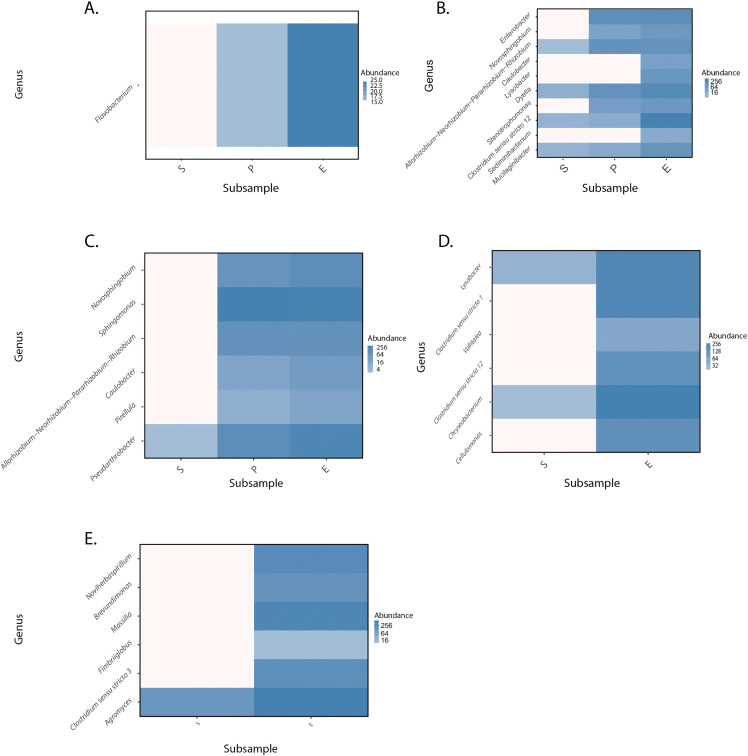
Heatmap of the cluster analysis by farm, using the ANCOM-BC analysis, with the most abundant bacteria at the genus level under treatment Cd added, extracted from the comparison with treatment without amendment. Where A = Farm1, B = Farm2, C = Farm3, D = Farm4, E = Farm5.

The result of the ANCOM-BC analysis revealed changes in taxa between the start, peak, and end of the experiments. *Flavobacterium* (Fig 6A) is here first reported as a new genus of Cd-tolerant bacteria in cacao soils. It was found that the heat produced in samples from farm 1 during the IMC experiment was related to the presence of the genus *Flavobacterium* **(arrow-** Fig 4A). It is worth mentioning that the time to the peak with of maximum heat production, is consistent with the time where aerobic metabolic reactions occur in the ampoule, and it is also related to the main ecological adaptation strategy of this genus [77]. Furthermore, some genera detected here have been previously reported for Cd-binding or tolerance [27,28]. In the same way, *Rhizobium* has been reported to have Cd-binding cell walls [78]*, Enterobacter* has demonstrated growth in Cd presence with *in vitro* assay conditions [28], and *Brevundimonas* is known for Cd biosorption [79].

Although several previously reported Cd-tolerant bacterial taxa were detected, it is important to acknowledge that microbial communities alone are unlikely to directly remove Cd contamination from soils. In this context, our focus was placed on bacterial taxa consistently detected across multiple farms, as their potential relevance lies in supporting Cd dynamics in soil rather than in direct metal removal. Genera such as *Novosphingobium, Caulobacter, Lysobacter, Rhizobium*, and *Clostridium sensu stricto 12* were identified in at least two of the five farms analyzed (Fig 6B–D), suggesting their persistence across different environmental conditions. These taxa may play indirect roles in Cd immobilization, mobilization, or bioavailability, potentially enhancing plant-based remediation strategies. Indeed, the use of suitable plant species for heavy metal removal has shown promise [80], and co-planting approaches in cacao agroecosystems could benefit from the interaction between Cd-tolerant bacteria and phytoremediation-capable plants. Analyzing the genera found in each thermogram performed, it was possible to point out the relationship between adaptive strategies of metabolic activity (aerobic or anaerobic reactions) and the relative abundance of genera found by sequencing. For instance, samples from farm 2 **(arrow in** Fig 4B**)**, exhibited maximum heat production after 40 hours of thermal monitoring. At such monitored time, oxygen concentrations begin to be limited in the ampoule, which agrees with the anaerobic metabolic strategy of the genera such as *Clostridium sensu stricto 12*. In contrast, samples from farm 3 **(left arrow in** Fig 4C**)** with the earliest peak of activity could be related to some genera such as *Sphingomonas, Rhizobium, Caulobacte*r, and *Pirellula,* all of them with strict or facultative aerobic metabolic requirements.

Furthermore, samples from farm 2 showed the most consistent increasable activity across the 80 hours of the experiment, which could be related to the high number of genera detected on those samples. One relevant genus found was *Enterobacter*, which was reported as a CdtB found in Santander cacao farms [28]. Other studies have supported the related genera here reported, with Cd tolerance. For instance, *Lysobacter* has been enriched to tolerate Cd, coming from Cd-contaminated soils [81]. Moreover, *Caulobacter*, an oligotrophic bacterium, has been isolated and mutated to test growth under Cd presence [82]. Likewise, *Novosphingobium* degrades a wide range of polycyclic aromatic hydrocarbons (PAHs) and Cd [83]. Interestingly, *Allo*-, *Neo-,* and *Para- Rhizobium* have been studied for their Cd-stress tolerance [84,85] under free-living facultative aerobic conditions, and some of their individuals produce polyhydroxyalkanoates (PHAs) useful for soil surveying during fixation stock carbon activities and decontamination process.

Therefore, the temporal sampling design of the IMC experiment enabled us to link taxonomic shifts with metabolic activity peaks and adaptive strategies. Genera showing the highest differential abundance during peak activity—including *Flavobacterium, Novosphingobium, Lysobacter*, and *Rhizobium*—possess established mechanisms for heavy metal tolerance and biosorption [61].

Taxa that responded under natural soil Cd conditions (S1 Table) differed from those detected under experimental Cd addition (S5 Fig). These differences are likely related to the controlled conditions of the experimental setup, which reduce environmental complexity and limit the influence of confounding factors. In contrast, soils with natural Cd content may be subject to additional environmental and historical processes that affect DNA persistence and detection, potentially introducing artifacts unrelated to current microbial activity. We acknowledge the limitations of the use of 16S rRNA as the only gene for assessing the bacterial community composition under the complexity of this kind of environment. At this point is critical to follow studies that measure the ecological role of these genera under natural conditions using functional genes related to Cd transformation (i.e., *cadA* or *smt* genes [27] and assess their competitiveness for potential bioaugmentation in cacao soils to remediate Cd and improve the cacao crop safety.

## Conclusions

This study applied a large-scale approach across contrasting cacao-growing environments to evaluate soil bacterial community responses to cadmium under natural and experimentally amended conditions using metabarcoding and isothermal microcalorimetry. Soil Cd concentrations were highly heterogeneous across and within farms, and a subset of bacterial taxa exhibited significant responses to Cd levels despite the complexity of environmental and agronomic factors. Although only a small proportion of taxa showed positive or negative associations with Cd, these responses were detectable at fine spatial scales. Bacterial metabolic activity and community composition varied according to farm location and changed over the course of the experiment, indicating dynamic community responses to environmental conditions. Several genera, including *Novosphingobium, Caulobacter, Lysobacter, Rhizobium*, and *Clostridium sensu stricto 12*, were consistently detected across multiple locations, some of which have been previously reported as Cd-tolerant. Overall, these findings underscore the value of integrating complementary approaches to characterize soil bacterial communities and represent a step forward in understanding bacterial responses to cadmium in agricultural soils.

## Supporting information

S1 Fig A. Venn diagram including all ASVs within each farm (unique ASVs) and between farms (shared ASVs). B. Venn diagram including all ASVs within each Cd-category (unique ASVs) and between Cd-categories (shared ASVs).
(PDF)

S2 Fig LDA showing the ANCOMBC result per phylum. Red color corresponds to the phylum with a positive response, comparing the Cd-categories (Cd_High_ vs. Cd_Low_). Blue color corresponds to the phylum with a negative response, comparing the Cd-categories (Cd_High_ vs. Cd_Low_).
(PDF)

S1 Table The ANCOMBC result shows the differential abundance of phyla that were detected in all the samples under natural conditions. The table shows phyla that had positive/negative differential abundance and phyla that didn´t respond to the Cd_soil_ as a factor evaluated.
(PDF)

S3 Fig A. Relative abundance of ASVs by phylum clustered by farms sampled (each number corresponds to farms) and B. the natural Cd_soil_ categories. (PDF)

S2 Table Thermodynamic parameters obtained by heat flow from IMC, fitting the Gompertz equation. The parameters were calculated to each sample clustered by agronomic unit.
(PDF)

S3 Table Summary of soil physico-chemical characteristics from the five farms. The average, minimum, and maximum of each parameter are presented in this table.
(PDF)

S4 Fig Canonical analysis of principal coordinates (CAP) of soil bacterial community composition in multivariate analysis with thermodynamic parameters obtained from isothermal microcalorimetry (IMC). The largest arrows indicate the major correlation between the variables and bacterial composition. The direction of the arrows indicates an increase in this variable, while variables pointing in the opposite direction indicate negative associations with community composition.
(PDF)

S5 Fig Phylum abundance in the samples evaluated during the experiment (Cd-responsive based on activity), including both with and without Cd amendment (i.e., control). Those with differential abundance are categorized as “positive response” and “negative response.” The phyla categorized as “non-responders” correspond to those with no significant change in abundance.
(PDF)
