# Supplementary material for: Integrating natural gradients and controlled assays to reveal bacterial responses to cadmium in Theobroma cacao L., soils
Source: PLoS One. 2026 Mar 24;21(3):e0345645. doi: 10.1371/journal.pone.0345645 (PMC13012491; doi:10.1371/journal.pone.0345645)
Supplement: S1 Fig — B. Venn diagram including all ASVs within each Cd-category (unique ASVs) and between Cd-categories (shared ASVs). (PDF) [file pone.0345645.s001.pdf]

A.

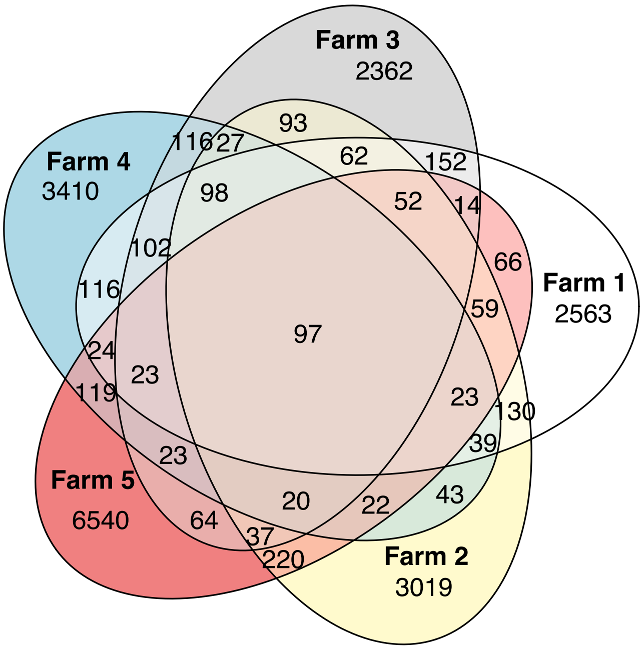

B.

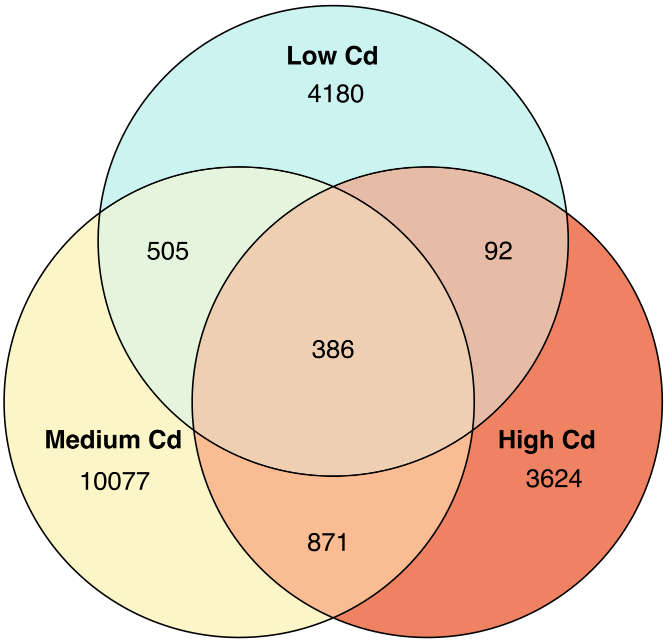

S1 Fig. *A*. Venn diagram including all ASVs within each farm (unique ASVs) and between farms (shared ASVs). ***B.*** Venn diagram including all ASVs within each Cd-category (unique ASVs) and between Cd-categories (shared ASVs).
