# Supplementary material for: Integrating natural gradients and controlled assays to reveal bacterial responses to cadmium in Theobroma cacao L., soils
Source: PLoS One. 2026 Mar 24;21(3):e0345645. doi: 10.1371/journal.pone.0345645 (PMC13012491; doi:10.1371/journal.pone.0345645)
Supplement: S2 Fig — Red color corresponds to the phylum with a positive response, comparing the Cd-categories (CdHigh vs. CdLow). Blue color corresponds to the phylum with a negative response, comparing the Cd-categories (CdHigh vs. CdLow). (PDF) [file pone.0345645.s002.pdf]

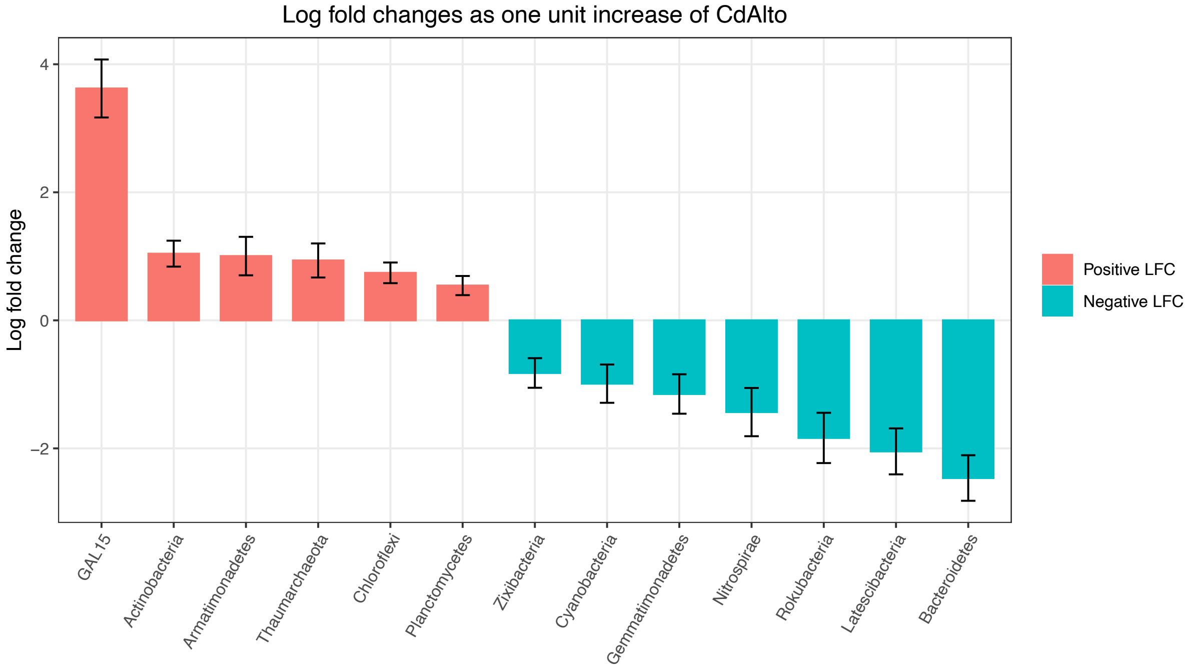

S2 Fig. LDA showing the ANCOMBC result per phylum. Red color corresponds to the phylum with a positive response, comparing the Cd-categories (Cd_{High} vs. Cd_{Low}). Blue color corresponds to the phylum with a negative response, comparing the Cd-categories (Cd_{High} vs. Cd_{Low}).
