# Supplementary material for: Integrating natural gradients and controlled assays to reveal bacterial responses to cadmium in Theobroma cacao L., soils
Source: PLoS One. 2026 Mar 24;21(3):e0345645. doi: 10.1371/journal.pone.0345645 (PMC13012491; doi:10.1371/journal.pone.0345645)
Supplement: S1 Table — The table shows phyla that had positive/negative differential abundance and phyla that didn´t respond to the Cdsoil as a factor evaluated. (PDF) [file pone.0345645.s003.pdf]

Positive differential abundance		***Negative differential abundance***	
Archaea	*Thaumarchaeota*	Bacteria	*Bacteroidetes*
Bacteria	*Armatimonadetes*	Bacteria	*Cyanobacteria*
Bacteria	*Actinobacteria*	Bacteria	*Gemmatimonadetes*
Bacteria	*Chloroflexi*	Bacteria	*Latescibacteria*
Bacteria	*GAL15*	Bacteria	*Nitrospirae*
Bacteria	*Planctomycetes*	Bacteria	*Rokubacteria*
-	-	Bacteria	*Zixibacteria*
Non-responders			
Archaea	*Crenarchaeota*	Bacteria	*Fibrobacteres*
Archaea	*Diapherotrites*	Bacteria	*Firmicutes*
Archaea	*Euryarchaeota*	Bacteria	*Hydrogenedentes*
Archaea	*Nanoarchaeaeota*	Bacteria	*Kiritimatiellaeota*
Bacteria	*Acidobacteria*	Bacteria	*Margulisbacteria*
Bacteria	*Aquificae*	Bacteria	*Omnitrophicaeota*
Bacteria	*BRC1*	Bacteria	*Patescibacteria*
Bacteria	*Calditrichaeota*	Bacteria	*Proteobacteria*
Bacteria	*Chlamydiae*	Bacteria	*Spirochaetes*
Bacteria	*Dadabacteria*	Bacteria	*Synergistetes*
Bacteria	*Dependentiae*	Bacteria	*Tenericutes*
Bacteria	*Dictyoglomi*	Bacteria	*Verrucomicrobia*
Bacteria	*Elusimicrobia*	Bacteria	*WPS-2*
Bacteria	*Entotheonellaeota*	Bacteria	*WS2*
Bacteria	*Epsilonbacteraeota*	Bacteria	*WS4*
Bacteria	*FBP*	unknown	*unknown*
Bacteria	*FCPU426*		

S1 Table. The ANCOMBC result shows the differential abundance of phyla that were detected in all the samples under natural conditions. The table shows phyla that had positive/negative differential abundance and phyla that didn ´t respond to the Cd_{soil} as a factor evaluated.
