# Supplementary material for: Integrating natural gradients and controlled assays to reveal bacterial responses to cadmium in Theobroma cacao L., soils
Source: PLoS One. 2026 Mar 24;21(3):e0345645. doi: 10.1371/journal.pone.0345645 (PMC13012491; doi:10.1371/journal.pone.0345645)
Supplement: S3 Fig — (PDF) [file pone.0345645.s004.pdf]

A.

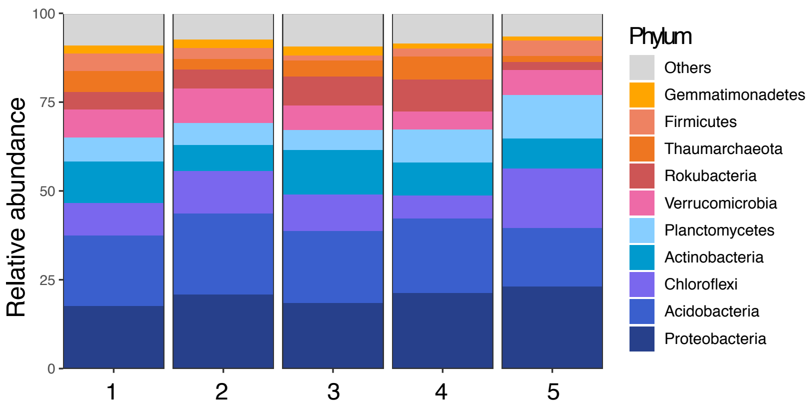

B.

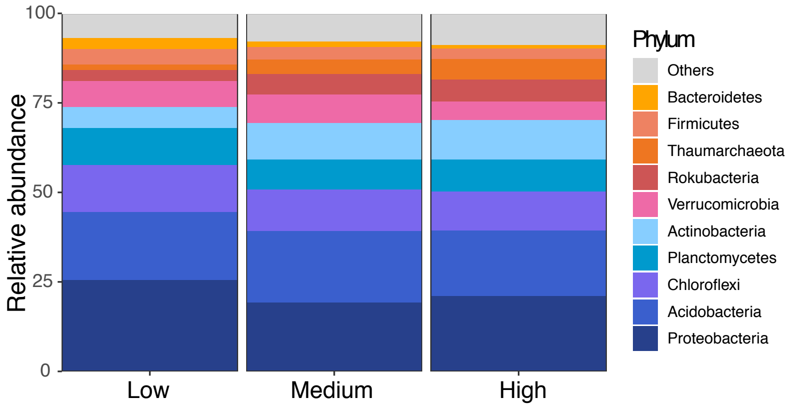

S3 Fig. (A) Relative abundance of ASVs by phylum clustered by farms sampled (each number corresponds to farms) and **(B)** the natural Cd_{soil} categories
