# Supplementary material for: Integrating natural gradients and controlled assays to reveal bacterial responses to cadmium in Theobroma cacao L., soils
Source: PLoS One. 2026 Mar 24;21(3):e0345645. doi: 10.1371/journal.pone.0345645 (PMC13012491; doi:10.1371/journal.pone.0345645)
Supplement: S2 Table — The parameters were calculated to each sample clustered by agronomic unit. (PDF) [file pone.0345645.s005.pdf]

**Farm 1**	*Natural Cd concentration (mg kg-1)*	*Heat flow [mW]*	*μ [h-1]*	*μmax [h-1]*	*λ [min]*	*Qmax [Joules]*	*p-value*
S01 (No amendment)<br>S01 (1 mg kg-1)	2.2	0.32<br>0.31	0.00051<br>0.0015	0.002<br>0.001	0<br>20.84	0.237<br>0.058	0.90<br>0.90
S02 (No amendment)<br>S02 (1 mg kg-1)	1.69	0.12<br>0.14	0.35<br>0.12	0.42<br>0.15	12.9<br>12.88	60.864<br>21.606	0.99<br>0.99
S03 (No amendment)<br>S03 (1 mg kg-1)	2.16	0.4<br>0.51	0.001<br>0.004	0.001<br>0.004	10.97<br>11.36	0.068<br>0.025	0.90<br>0.90
S04 (No amendment)<br>S04 (1 mg kg-1)	1.85	0.49<br>0.3	0.0008<br>0.0007	0.001<br>0.001	0<br>3.5	0.075<br>0.05	0.90<br>0.90
S05 (No amendment)<br>S05 (1 mg kg-1)	1.70	0.5<br>0.35	0.0006<br>0.0006	0.001<br>0.001	10.36<br>16.87	0.04<br>0.039	0.90<br>0.90
S06 (No amendment)<br>S06 (1 mg kg-1)	1.71	0.11<br>0.21	0.03<br>0.3	0.035<br>0.37	12.49<br>12.75	5.132<br>53.354	0.99<br>0.99

**Farm2**	*Natural Cd concentration (mg kg^{-1})*	*Heat flow [mW]*	*μ [h-1]*	*μ max [h-1]*	*λ [min]*	*Q_{max} [Joules]*	*p-value*
S07 (No amendment)<br>S07 (1 mg kg^{-1})	1.6	1.2<br>2	0.003<br>0.006	0.004<br>0.006	23.06<br>29.76	0.244<br>0.342	0.998<br>0.998
S08 (No amendment)<br>S08 (1 mg kg^{-1})	1.64	1.5<br>1.4	0.004<br>0.004	0.004<br>0.004	23.31<br>23.96	0.279<br>0.274	0.998<br>0.998
S09 (No amendment)<br>S09 (1 mg kg^{-1})	1.45	148<br>120	0.361<br>0.309	0.464<br>0.464	4.55<br>4.55	24.65<br>24.66	0.993<br>0.993
S10 (No amendment)<br>S10 (1 mg kg^{-1})	1.5	90<br>91	0.219<br>0.208	0.28<br>0.267	4.63<br>4.53	14.88<br>14.21	0.993<br>0.993
S11 (No amendment)<br>S11 (1 mg kg^{-1})	1.48	33<br>20	0.064<br>0.037	0.063<br>0.036	21.85<br>21.53	2.812<br>1.63	0.993<br>0.993
S12 (No amendment)<br>S12 (1 mg kg^{-1})	1.39	39<br>13	0.089<br>0.027	0.109<br>0.026	5.84<br>21.86	5.865<br>1.159	0.993<br>0.993

**Farm3**	*Natural Cd concentration (mg kg^{-1})*	*Heat flow [mW]*	*μ [h-1]*	*μ max [h-1]*	*λ [min]*	*Q_{max} [Joules]*	*p-value*
S13 (No amendment)<br>S13 (1 mg kg^{-1})	2.08	242<br>310	0.009<br>0.474	0.016<br>0.613	2.66<br>44.23	0.737<br>0.193	0.991<br>0.991
S14 (No amendment)<br>S14 (1 mg kg^{-1})	1.82	0.9<br>8	0.003<br>0.024	0.003<br>0.02	17.59<br>38.54	0.212<br>1.107	0.998<br>0.998
S15 (No amendment)<br>S15 (1 mg kg^{-1})	1.94	0.7<br>242	0.001<br>0.393	0.001<br>0.267	9.10<br>43.98	0.069<br>0.157	0.991<br>0.991
S16 (No amendment)<br>S16 (1 mg kg^{-1})	2.03	238<br>0.48	0.331<br>0.001	0.040<br>0.001	41.8<br>4.69	0.163<br>0.022	0.991<br>0.991
S17 (No amendment)<br>S17 (1 mg kg^{-1})	1.81	1.12<br>1.3	0.003<br>0.003	0.003<br>0.003	22.08<br>20.37	0.227<br>0.233	0.998<br>0.998
S18 (No amendment)<br>S18 (1 mg kg^{-1})	2.17	0.4<br>0.32	0.001<br>0.001	0.001<br>0.001	12.01<br>12.16	0.042<br>0.046	0.991<br>0.991

**Farm4**	*Natural Cd concentration (mg kg-^{1})*	*Heat flow [mW]*	*μ [h-1]*	*μ max [h-1]*	*λ [min]*	*Qmax [Joules]*	*p-value*
S19 (No amendment)<br>S19 (1 mg kg^{-1})	3.05	48<br>27	0.13<br>0.07	0.18<br>0.10	1.86<br>1.36	9.521<br>5.321	0.998<br>0.998
S20 (No amendment)<br>S20 (1 mg kg^{-1})	3.13	28<br>68	0.08<br>0.18	0.11<br>0.26	2.06<br>1.97	5.77<br>13.618	0.998<br>0.998
S21 (No amendment)<br>S21 (1 mg kg^{-1})	1.90	50<br>29	0.14<br>0.08	0.20<br>0.11	2.03<br>1.96	10.189<br>5.727	0.998<br>0.998
S22 (No amendment)<br>S22 (1 mg kg^{-1})	2.01	27<br>81	0.08<br>0.23	0.11<br>0.32	1.88<br>1.99	5.684<br>16.753	0.998<br>0.998
S23 (No amendment)<br>S23 (1 mg kg^{-1})	3.09	3<br>26	0.01<br>0.004	0.01<br>0.006	29.95<br>15.79	1<br>0.336	0.997<br>0.997
S24 (No amendment)<br>S24 (1 mg kg^{-1})	1.98	1.6<br>3	0.005<br>0.01	0.006<br>0.01	10.75<br>13.53	0.542<br>0.709	0.997<br>0.997

Farm5	*Natural Cd concentration (mg kg^{-1})*	*Heat flow [mW]*	*μ [h-1]*	*μ max [h-1]*	*λ [min]*	*Q_{max} [Joules]*	*p-value*
S25 (No amendment)	0.89	2.9	0.008	0.009	17.97	0.568	0.997
S25 (1 mg kg^{-1})		1.6	0.004	0.004	12.67	0.324	0.997
S26 (No amendment)	0.87	2	0.001	0.001	22.79	0.158	0.970
S26 (1 mg kg^{-1})		1.3	0.003	0.003	15.84	0.426	0.970
S27 (No amendment)	0.34	1.2	0.002	0.002	16.85	0.328	0.970
S27 (1 mg kg^{-1})		2.5	0.004	0.005	11.76	0.774	0.970
S28 (No amendment)	0.42	8.4	0.002	0.002	13.04	0.226	0.997
S28 (1 mg kg^{-1})		5	0.01	0.013	19.56	0.842	0.997
S29 (No amendment)	0.73	2.7	0.004	0.006	9.16	0.791	0.970
S29 (1 mg kg^{-1})		1.4	0.002	0.002	5.98	0.323	0.970
S30 (No amendment)	0.18	0.7	0.001	0.002	8.37	0.221	0.970
S30 (1 mg kg^{-1})		2.7	0.004	0.005	20.54	0.718	0.970

S2 Table. Thermodynamic parameters obtained by heat flow from IMC, fitting the Gompertz equation. The parameters were calculated for each sample clustered by agronomic unit.
