# Supplementary material for: Integrating natural gradients and controlled assays to reveal bacterial responses to cadmium in Theobroma cacao L., soils
Source: PLoS One. 2026 Mar 24;21(3):e0345645. doi: 10.1371/journal.pone.0345645 (PMC13012491; doi:10.1371/journal.pone.0345645)
Supplement: S3 Table — The average, minimum, and maximum of each parameter are presented in this table. (PDF) [file pone.0345645.s006.pdf]

	FARMS					
Physicochemical parameters	1	2	3	4	5	Grand Total
Average of CCS	1.78	1.57	1.83	2.21	0.96	1.57
Min. of CCS	1.62	1.45	1.83	1.87	0.41	0.41
Max. of CCS	1.93	1.77	1.84	2.76	1.58	2.76
Average of CCF	15.67	1.33	1.29	8.95	4.19	6.34
Min. of CCF	14.78	1.20	0.78	5.58	0.33	0.33
Max. of CCF	16.86	1.45	1.81	11.52	8.06	16.86
Average of CCDisS	0.27	0.15	0.34	0.69	0.06	0.27
Min. of CCDisS	0.15	0.13	0.30	0.53	0.01	0.01
Max. of CCDisS	0.35	0.17	0.39	0.94	0.11	0.94
Average of pH	5.52	5.70	6.19	6.73	6.30	6.11
Min. of pH	5.00	5.10	6.19	6.50	4.70	4.70
Max. of pH	6.36	6.30	6.19	7.00	7.20	7.20
Average of CARBONO	1.40	1.00	1.30	2.66	1.36	1.54
Min. of CARBONO	1.27	0.74	1.30	2.32	0.64	0.64
Max. of CARBONO	1.48	1.14	1.30	2.85	1.76	2.85
Average of P	6.67	10.47	3.87	31.47	56.74	27.33
Min. of P	3.08	5.25	3.87	10.30	12.50	3.08
Max. of P	9.15	13.50	3.87	46.20	102.00	102.00
Average of Ca	24.45	12.04	22.51	25.50	6.64	16.51
Min. of Ca	18.40	7.61	22.51	23.10	1.67	1.67
Max. of Ca	28.94	15.40	22.51	27.20	9.28	28.94
Average of Mg	5.76	5.34	7.36	1.68	1.02	3.63
Min. of Mg	4.99	5.02	7.36	1.26	0.29	0.29
Max. of Mg	6.92	5.66	7.36	2.03	1.44	7.36
Average of Na	0.26	0.17	0.14	0.03	0.06	0.12
Min. of Na	0.19	0.13	0.14	0.02	0.01	0.01
Max. of Na	0.30	0.23	0.14	0.03	0.11	0.30
Average of K	0.81	0.53	1.01	0.38	0.13	0.49
Min. of K	0.75	0.42	1.01	0.31	0.09	0.09
Max. of K	0.89	0.69	1.01	0.43	0.15	1.01
Average of B	0.26	0.27	0.26	0.06	0.19	0.20
Min. of B	0.12	0.20	0.26	0.04	0.04	0.04
Max. of B	0.44	0.39	0.26	0.09	0.32	0.44
Average of Fe	43.15	58.41	19.24	32.10	24.59	35.15
Min. of Fe	28.06	54.10	19.24	28.60	3.76	3.76
Max. of Fe	61.80	65.72	19.24	37.20	51.10	65.72
Average of Mn	9.30	9.22	1.97	8.35	2.16	5.96
Min. of Mn	2.04	2.53	1.97	7.14	0.66	0.66
Max. of Mn	15.90	18.00	1.97	9.42	5.89	18.00
Average of Cu	2.82	2.78	2.59	0.85	0.70	1.75
Min. of Cu	2.31	1.91	2.59	0.68	0.38	0.38
Max. of Cu	3.44	3.90	2.59	1.11	0.98	3.90
Average of Zn	1.63	1.26	1.63	4.64	4.32	2.97
Min. of Zn	1.08	1.00	1.63	1.86	0.41	0.41
Max. of Zn	2.01	1.46	1.63	6.73	10.50	10.50
Average of S	9.71	27.63	4.44	9.49	14.07	13.73
Min. of S	4.68	15.39	4.44	4.68	2.84	2.84
Max. of S	14.20	51.80	4.44	13.10	29.70	51.80
Average of CIC	37.60	23.60	30.98	33.80	8.92	24.47
Min. of CIC	32.80	19.00	30.98	31.80	4.40	4.40
Max. of CIC	43.20	31.20	30.98	37.40	11.80	43.20
Average of CE	0.16	0.16	0.26	0.12	0.09	0.14
Min. of CE	0.10	0.08	0.26	0.09	0.07	0.07
Max. of CE	0.24	0.23	0.26	0.15	0.13	0.26

S3 Table. Summary of soil physico-chemical characteristics from the five farms. The average, minimum, and maximum of each parameter are presented in this table.
