# Supplementary material for: Integrating natural gradients and controlled assays to reveal bacterial responses to cadmium in Theobroma cacao L., soils
Source: PLoS One. 2026 Mar 24;21(3):e0345645. doi: 10.1371/journal.pone.0345645 (PMC13012491; doi:10.1371/journal.pone.0345645)
Supplement: S4 Fig — The largest arrows indicate the major correlation between the variables and bacterial composition. The direction of the arrows indicates an increase in this variable, while variables pointing in the opposite direction indicate negative associations with community composition. (PDF) [file pone.0345645.s007.pdf]

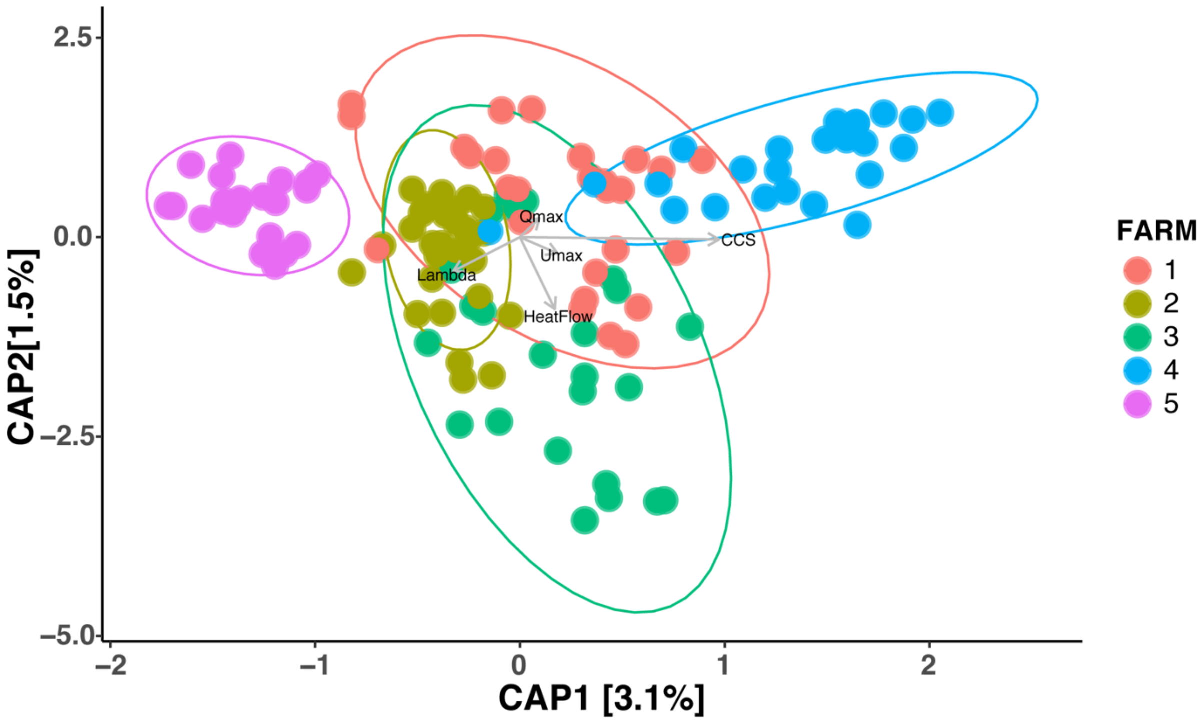

S4 Fig. Canonical analysis of principal coordinates (CAP) of soil bacterial community composition in multivariate analysis with thermodynamic parameters obtained from isothermal microcalorimetry (IMC). The largest arrows indicate the major correlation between the variables and bacterial composition. The direction of the arrows indicates an increase in this variable, and the opposite site corresponds to a decrease.
