# Supplementary material for: Integrating natural gradients and controlled assays to reveal bacterial responses to cadmium in Theobroma cacao L., soils
Source: PLoS One. 2026 Mar 24;21(3):e0345645. doi: 10.1371/journal.pone.0345645 (PMC13012491; doi:10.1371/journal.pone.0345645)
Supplement: S5 Fig — Those with differential abundance are categorized as “positive response” and “negative response.” The phyla categorized as “non-responders” correspond to those with no significant change in abundance. (PDF) [file pone.0345645.s008.pdf]

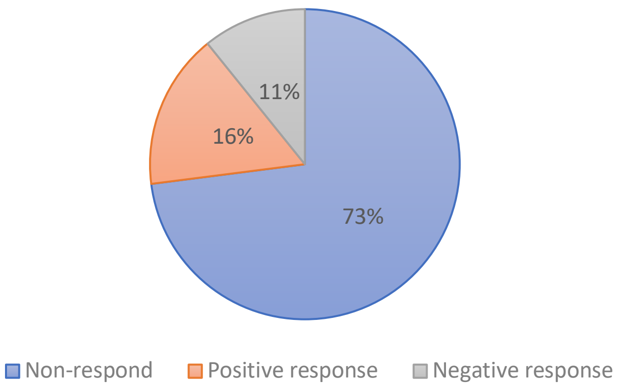

Positive response
Firmicutes
Bacteroidetes
Patescibacteria
Proteobacteria
Cyanobacteria
Actinobacteria

Negative response
Chloroflexi
Acidobacteria
Planctomycetes
Nitrospirae

Non-responders	
Crenarchaeota	*FCPU426*
Euryarchaeota	*Fibrobacteres*
Nanoarchaeaeota	*Firmicutes*
Thaumarchaeota	*GAL15*
Acidobacteria	*Gemmatimonadetes*
Actinobacteria	*Hydrogenedentes*
Armatimonadetes	*Latescibacteria*
Bacteroidetes	*Nitrospirae*
BRC1	*Omnitrophicaeota*
Calditrichaeota	*Patescibacteria*
Chlamydiae	*Planctomycetes*
Chloroflexi	*Proteobacteria*
Cyanobacteria	*Rokubacteria*
Dadabacteria	*Verrucomicrobia*
Deinococcus-Thermus	*WPS-2*
Dependentiae	*WS2*
Elusimicrobia	*WS4*
Entotheonellaeota	*Unknown*
FBP	

S5 Fig. Phylum in the samples evaluated during the experiment (Cd-response based on activity), including both with and without Cd amendment (i.e., control). Those with differential abundance are categorized as “positive response” and “negative response”. The phyla categorized as “non-responders” correspond to those with no significant changes in abundance.
